# Impact of bowel preparation on caecal intubation time during colonoscopy

**DOI:** 10.12669/pjms.35.6.1031

**Published:** 2019

**Authors:** Haris Alvi, Tazeen Rasheed, Majid Ahmed Shaikh, Faiza Sadaqat Ali, Baber Faiyaz Zuberi, Asad Ali Samejo

**Affiliations:** 1 Prof. Haris Alvi, MBBS, FCPS. Dow University of Health Sciences, Karachi, Pakistan; 2 Dr. Tazeen Rasheed Assistant Professor, Dow University of Health Sciences, Karachi, Pakistan; 3 Dr. Majid Ahmed Shaikh Assistant Professor, Dow University of Health Sciences, Karachi, Pakistan; 4 Dr. Faiza Sadaqat Ali Senior Registrar, Dow University of Health Sciences, Karachi, Pakistan; 5 Prof. Bader Faiyaz Zuberi, Dow University of Health Sciences, Karachi, Pakistan; 6 Dr. Asad Ali Samejo Postgraduate Trainee, Dow University of Health Sciences, Karachi, Pakistan

**Keywords:** Boston bowel preparation scale, Colonoscopy, Caecal intubation time, Adenoma Detection Rate

## Abstract

**Objective:**

To determine the caecal intubation time depending on bowel preparation as per Boston Bowel Preparation Scale.

**Methods:**

This cross-sectional study was conducted at Dr. Ruth K. M. Pfau, Civil Hospital Karachi between August 2018 to February 2019. A total of 201 patients were included in the study. Time was recorded from insertion of colonoscope to the time required to reach the cecum. Bowel preparation was graded during withdrawal of colonoscope by using Boston Bowel Preparation Scale. Pearson Correlation test was used to study correlation of BBPS scores with CIT, gender, BMI, adenoma and polyp detection.

**Results:**

In this study 201 patients undergoing colonoscopy were included. Mean ±SD of age of patients was 36.9 ±15.8 years. Out of the 201 patients 112 (56%) were males and 89 (44%) were females. The results of our study showed that increased Boston Bowel Preparation Scale Scores were associated with decreased caecal intubation time. The mean CIT was 10.7 ±5.4 minutes and Pearson correlation was significant at 0.002. Significant correlations of BBPS were also found with BMI and adenoma detection.

**Conclusion:**

The diagnostic effectiveness of colonoscopy depends upon the quality of the preparation. Good bowel preparation improves the speed of colonoscopy and its completeness.

## INTRODUCTION

Caecal intubation time is defined as the time required to reach caecum after insertion of colonoscope. It indicates the difficulty of advancing the scope till caecum.^1^ It is affected by many factors including age, sex, increased or decreased body mass index, waist circumference, history of abdominal or pelvic surgery, experience of endoscopist, and the bowel preparation.^1^-^3^ It is also affected by length of colonoscope and advance features present in colonoscope such as variable stiffness.^4^ Although other factors are measurable and obtained from history, but bowel preparation is measured by different scales including American Society for Gastrointestinal Endoscopy (ASGE) and American Gastroenterological Association (AGA) Task Force who used the terms as “excellent, good, fair and poor”. This lacks standard definition and results in variability in comments on bowel preparation.^5^,^6^

Another scale named as Boston Bowel Preparation Scale (BBPS) is considered as a novel scale for rating of bowel cleanliness has received good intra and inter-observer reliability assessments and is designed for researches related to colonoscopy and bowel preparation.^7^,^8^ It assesses the bowel during withdrawal after washing and suctioning.^8^ BBPS scores correlates with polyp detection rates and caecal intubation time.^5^,^8^ Lower BBPS score is associated with missed polyp detection and requires early repeat colonoscopy as it has negative impact on colon cancer prevention.^6^,^9^

Our objective was to determine the impact of bowel preparation on caecal intubation time, as good bowel preparation will lead to shorter caecal intubation time, better visualization of mucosa and polyp detection.

## METHODS

This cross-sectional study was conducted at Dr. Ruth K. M. Pfau, Civil Hospital Karachi between August 2018 to February 2019. Non-probability consecutive sampling was used for selection of patients. Approval was taken from the Institutional Review Board of Dow University of Health Sciences.

### Inclusion criteria

All patients between the ages of 18-70 years undergoing colonoscopy were included. Exclusion criteria: Patients who were unable to tolerate colonoscopy preparation solutions and patients having electrolyte abnormalities to whom colonoscopy preparation solution could not be given, were excluded.

A written informed consent was taken from the patients undergoing colonoscopy. Patients were advised to take colonoscopy preparation solution at 12 PM then at 6 PM the day before colonoscopy. They were advised to take liquid diet for 24 hours before colonoscopy and nil oral orders for four hours before colonoscopy. Each colonoscopy preparation kit contains two 45 ml bottles of solution. Each 45 ml bottle contains: sodium sulfate 17.5 grams, potassium sulfate 3.13 grams, magnesium sulfate 1.6 grams. Inactive ingredients include sodium benzoate NF, sucralose, malic acid FCC, citric acid USP, flavoring ingredients, purified water, USP. The solution is diluted to a final volume of 500 ml with water before intake. Colonoscopy were done by three skilled colonoscopists which in study were called as A, B & C. Time was recorded from insertion of colonoscope in anus till caecum is reached. Bowel preparation was graded during withdrawal of colonoscope by using BBPS from 0 to 3, where higher score means good bowel preparation, details of Boston Bowel Scale is given as under:

0 = poor preparation with solid stool on mucosa that cannot be cleaned and hinder the mucosal visualization.
1 = some mucosa seen while rest of mucosa cannot be seen due to solid or loose stool.
2 = some mucosa cannot be seen due to solid or loose stool while rest of mucosa is visualized.
3 = good preparation and visualization of entire mucosa.^7^

It is the sum of three individual segments scale.

First Segment Caecum & Ascending Colon

Second Segment Transverse Colon with Hepatic & Splenic Flexures

Third Segment Descending, Sigmoid Colon & Rectum

The impact of bowel preparation on caecal intubation time was analyzed separately for individual colonoscopists as well as combined for all three too. BBPS scores was correlated with CIT, gender, BMI, adenoma and polyp detection using ‘Pearson Correlation Test’. Value of ≤0.05 was taken as significant.

## RESULTS

In this study 201 patients undergoing colonoscopy were included. Three colonoscopists from our department participated in this study. Out of the 201 colonoscopies, 72 were performed by Colonoscopist-A, 35 by Colonoscopist-B and 94 by Colonoscopist-C. Mean ±SD of age of patients was 36.9 ±15.8 years. Out of the 201 patients 112 (56%) were males and 89 (44%) were females. Mean age of males was 34.8 ±16.6 years while that of females was 39.6 ±14.5 years. The difference in age among gender was statistically significant (p = 0.03; df 199; 95% CI -9.2 to -0.4). Mean BMI of patients undergoing colonoscopy was 24.2 ±3.5. Mean BMI of female was significantly more as compared to males (p = 0.001; df 199; 95% CI -0.48 to -2.57). Mean caecal intubation time was 10.7 ±5.4 minutes. In males it was 10.4 ±5.2 minutes while in females it was 11.2 ±5.6 minutes. Difference between caecal intubation time in males and females was not significant (p = 0.27; df 199; 95% CI -2.4 to 0.7). Details are given in Table-I.

**Table I:** Details of Age, BMI & Caecal Time Comparison with Gender and P-values.

	Total Mean ±SD	Males Mean ±SD	Female Mean ±SD	P Value T-Test^1^
Age (years)	36.9 ±15.8	34.8 ±16.6	39.6 ±14.5	0.031
BMI	24.3 ±3.5	23.5 ±3.4	25.2 ±3.5	0.001
Caecal Time (minutes)	10.7 ±5.4	10.4 ±5.2	11.2 ±5.6	0.276

1 Significant Level ≤0.05.

A total of four adenomas were detected by Colonoscopist-A whereas no adenomas were detected by Colonoscopist-B and C. A total of 29 polyps were detected out of which 14 (48.3%) polyps were detected by Colonoscopist-A, 3 (10.3%) by Colonoscopist-B and 12 (41.4%) by Colonoscopist-C.

The caecal intubation rate was 100% by all three colonoscopists. The mean CIT of Colonoscopist-A was 9.2±4.2 minutes, Colonoscopist-B was 14.9±6.6 minutes and of Colonoscopist-C was 10.4±5.0 minutes. Difference in caecal intubation time was significantly different among the three colonoscopists when tested with ANNOVA (P <0.001). The correlation of CIT with BBPS by all three colonoscopists combined showed strong correlation with two tailed correlation at 0.002. Individual colonoscopists caecal intubation times also showed strong correlation with BBPS scores, details are given in Table-II. No correlation of CIT was found with BMI (p = 0.479). Adenoma detection rates also correlated significantly with BBPS scores (p < 0.001) but polyp detection was not found to correlate with BBPS scores (p = 0.173)

**Table II:** Caecal Intubation Times of Colonoscopists & Correlation with BBPS.

Colonoscopist	CIT^†^ (minutes)	P Value by ANNOVA^‡^	Correlation^§^ CIT & BBPS**
A	9.2 ±4.2	<0.001	<0.001
B	14.9 ±6.6		0.041
C	10.4 ±5.0		0.009
Mean of all 3	10.7 ±5.4		0.002

† CIT: Caecal Intubation Time,

‡ Significant Level ≤0.05

§ Pearson Correlation 2 Tailed,

** BBPS: Boston Bowel Preparation Scale

## DISCUSSION

The BBPS has been validated in a number of clinical trials.^5^,^10^ It was developed in 2009 and was designed to specify the issues affecting bowel preparation. The potential benefit of colonoscopy can only be achieved if the procedure is completed safely in minimum time with good visualization of the mucosa. Colonoscopy is widely used nowadays for therapeutic and diagnostic purposes. Its effectiveness is highly dependent on the quality of bowel preparation. Caecal intubation time is defined as the time required to reach caecum after insertion of colonoscope. Till now, the BBPS is considered the most reliable and the most relevant bowel preparation scale. It is also a simple scoring system that can be used in clinical routine practice.^11^ The aim of colonoscopy is to visualize the entire colon and skilled colonoscopists should intubate the caecum in at least 90% of patients.^12^

In our study we aimed to determine CIT &, rate and correlate the CIT with BBPS score. Secondary objective was to see adenoma detection rate and correlation of BBPS score with BMI. The results of our study showed that increased BBPS score, i.e., good colon preparation was associated with short CIT. The result of our study corresponded with the earlier studies where mean caecal intubation time was found to be shorter with increased BBPS score.^6^ As consistent with the previous studies this study also highlighted the validity and reliability of BBPS. Jang JY in his study reported that poor bowel preparation increases the overall procedure time, reduces the caecal intubation rate, increases the costs of colonoscopy and increases chances of missing polyps or adenomas during colonoscopy.^13^ In our study adenoma detection rate was also associated with good bowel preparation but it was also linked with the skills and experience of colonoscopists.

Caecal intubation was considered successful if caecal landmarks (caecal strap fold and ileo-caecal valve) were photo-documented. Bowles C *et al*. in their study reported 5251/9223, i.e., 56.9% objectively confirmed complete colonoscopies and in 1913/9223 (20.7%) procedures the endoscopist was unable to complete the colonoscopy.^14^ According to them the most common cause of this high failure rate was patient discomfort (34.7%) followed by uncontrolled looping (29.7%), poor bowel preparation (19.6%), diverticulosis (9.5%), adequate delineation of subtotal colitis (2.0%), resected caecum (7.2%) and tumor in proximal colon (5.6%).^14^ They highlighted that one out of five incomplete colonoscopies were caused by suboptimal bowel preparation. In contrast to their result, in our study, caecal intubation rate by all three colonoscopist were 100%. Although we found slight difference in mean caecal intubation time. This difference was probably due to skills and experience difference among three colonoscopists. While Akere A et al. in his study on 167 patients showed that increased BMI had negative impact on CIT but in our study we did not find any association of CIT with BMI.^15^

### Limitation of the study

As the colonoscopies were performed by three colonoscopist so difference in their duration of experience and technique may contribute to CIT. But we try to minimize this confounder by assessing mean CIT with BBPS by all three colonoscopist separately also. Another limitation of this study was that patient’s pain tolerance was not assessed prior to colonoscopy which may also contribute to the speed of colonoscopy. We used sedation and analgesia prior to every individual colonoscopy to minimize this difference in pain threshold. Another limitation of this study was that it was done in single center.

## CONCLUSION

The diagnostic effectiveness of colonoscopy depends upon the quality of the preparation. This study showed that good bowel preparation improves the speed of colonoscopy, its completeness, rate of polyp and adenoma detection.

### Authors’ Contribution:

**HA:** Did coloscopies, Study Conception.

**TR:** Data collection, initial manuscript writing.

**MAS:** Did colonoscopies, manuscript review and statistical analysis.

**FSA:** Manuscript writing and statistical analysis.

**BFZ:** Did colonoscopies, final corrections and approval of manuscript, is responsible for integrity of research.

**AAS:** Data collection, initial manuscript writing.
